# Plant Fibre: Molecular Structure and Biomechanical Properties, of a Complex Living Material, Influencing Its Deconstruction towards a Biobased Composite

**DOI:** 10.3390/ma9080618

**Published:** 2016-07-26

**Authors:** Mathias Sorieul, Alan Dickson, Stefan J. Hill, Hamish Pearson

**Affiliations:** Scion, Private Bag 3020, Rotorua 3046, New Zealand; Alan.dickson@scionresearch.com (A.D.); Stefan.hill@scionresearch.com (S.J.H.); Hamish.pearson@scionresearch.com (H.P.)

**Keywords:** biological material, plant fibre, cell wall, hemicellulose, molecular interactions, reinforced plastics, biobased composites

## Abstract

Plant cell walls form an organic complex composite material that fulfils various functions. The hierarchical structure of this material is generated from the integration of its elementary components. This review provides an overview of wood as a composite material followed by its deconstruction into fibres that can then be incorporated into biobased composites. Firstly, the fibres are defined, and their various origins are discussed. Then, the organisation of cell walls and their components are described. The emphasis is on the molecular interactions of the cellulose microfibrils, lignin and hemicelluloses *in planta*. Hemicelluloses of diverse species and cell walls are described. Details of their organisation in the primary cell wall are provided, as understanding of the role of hemicellulose has recently evolved and is likely to affect our perception and future study of their secondary cell wall homologs. The importance of the presence of water on wood mechanical properties is also discussed. These sections provide the basis for understanding the molecular arrangements and interactions of the components and how they influence changes in fibre properties once isolated. A range of pulping processes can be used to individualise wood fibres, but these can cause damage to the fibres. Therefore, issues relating to fibre production are discussed along with the dispersion of wood fibres during extrusion. The final section explores various ways to improve fibres obtained from wood.

## 1. Introduction

Natural fibres of various types have been essential to mankind for centuries. Plastics and synthetic fibres invented during the 20th century have led to the generation of numerous types of synthetic fibre-reinforced polymer composites that are now widely used in everyday life. However, such synthetic materials are mostly generated from non-renewable sources, so the use of plant fibres in composites (known as biobased composites) is gaining interest. This is due not only to their eco-friendly and sustainable aspects, but more importantly, to their performance and cost.

The advantages of using natural, rather than synthetic, fibres in composites are numerous (but some may depend on the resin used): low specific weight; safer handling; not abrasive to mixing and moulding equipment; high electrical resistance; recyclable and/or recoverable; and good acoustic insulation (due to their hollow cellular structure). A wide diversity of plant species can be used to produce fibres suitable for use in industrial applications, but their morphology and mechanical properties will vary depending on their origin [1,2]. However, natural fibres do have major drawbacks. The anisotropic nature of natural fibres [3,4] is one issue, as is their hydrophilic nature, which affects their hygroscopic behaviour and ageing [5,6]. Water absorption induces swelling and can result in the presence of voids at the interface with the matrix and accelerates fibre aging. This results in poor mechanical properties and reduces the dimensional stability of the final composites. Natural fibre composites also suffer from: inferior fire resistance; lower mechanical properties; non-uniform dispersion; degradation when heated over 200 °C; and greater susceptibility to rotting compared to synthetic fibre ones. Some of the drawbacks of using cellulose-based, natural fibres as reinforcements of plastics (thermoset (irreversible cross-linking) and thermoplastics (possible remodelling when heated)) can be addressed by a wide range of chemical treatments [7,8,9].

**Figure 1:**
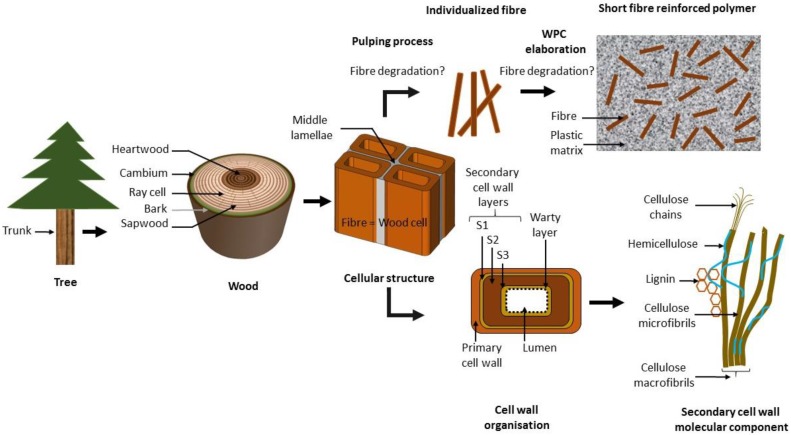
Overview of wood; from a living tree to the molecular organisation and its deconstruction to produce a short fibre-reinforced polymer composite. WPC, wood polymer composite. This figure outlines the topics covered by this review: the organisation of the aerial part of a tree and its trunk; the arrangement of the fibres in the wood; the structure of the cell wall and its molecular organisation; the deconstruction of the wood, fibre individualisation and integration into a WPC. This scheme is not pertinent for monocotyledons.

The primary aim of this review is to assist researchers intending to use cellulosic fibres, and mainly wood fibres, as a component in a short fibre-reinforced polymer material, also known as a wood polymer composite (WPC). We focus on the biological origin of wood fibre properties and describe how their functions of support and sap conduction define their hierarchically-organised architecture and molecular interactions in both primary and secondary cell walls. This approach has been undertaken because exhaustive overviews of cell wall composition and architecture are relatively rare, as they are usually restricted to either primary or secondary cell walls. We then discuss how the use of various wood pulping processes to individualise the fibres affects their mechanical properties. We finish by describing how various types of processing affect the performance of fibres once they are amalgamated into a WPC. The key steps of this approach are illustrated in Figure 1.

## 2. Wood

### 2.1. Wood Function

Wood is a complex composite material that fulfils a range of functions. The roles of wood, in a living tree, are to support the aerial part, transport water and store carbohydrates [10]. The bark is external to the wood. Beneath the bark lies the vascular cambium, which is composed of a few layers of living cells that actively divide and produce more wood, which results in an increase in the girth of the tree (Figure 1). Wood may be sub-divided into sapwood and heartwood. Sapwood conducts water and inorganic nutrients (sap), while heartwood is old sapwood that and has been chemically changed by the accumulation of resins and extractives, leading to the loss of water conductance properties. Trees may be divided into two main categories; ‘hardwoods’ and ‘softwoods’. Hardwoods are angiosperms (flowering plants), and softwoods are gymnosperms (evergreen, conifers).

### 2.2. Wood as a Composite Material

The concept of wood as a naturally-occurring bio-polymeric fibre-reinforced matrix was introduced in the 1920s by Ambronn and Frey [11], and has been regarded and extensively mathematically modelled as such since [12,13,14,15,16,17,18,19,20,21,22,23,24,25]. From a composites perspective, a ‘fibre’ is a material with a relatively high aspect ratio (≥10) of length to thickness that provides a discernible reinforcing effect. The macrostructure of solid wood can be classified into fibre-reinforced matrix models at two scales. At the microstructural level, the fibres (cell walls) are often modelled as a regular array of structural elements in a binding matrix (middle lamella). At the ultrastructural level, microfibril bundles of cellulose are embedded in an isotropic matrix (lignin and hemicellulose) [26,27]. At both levels, wood is a highly hierarchically engineered and structured material.

## 3. Fibre

### 3.1. Definition of Fibre

**Figure 2:**
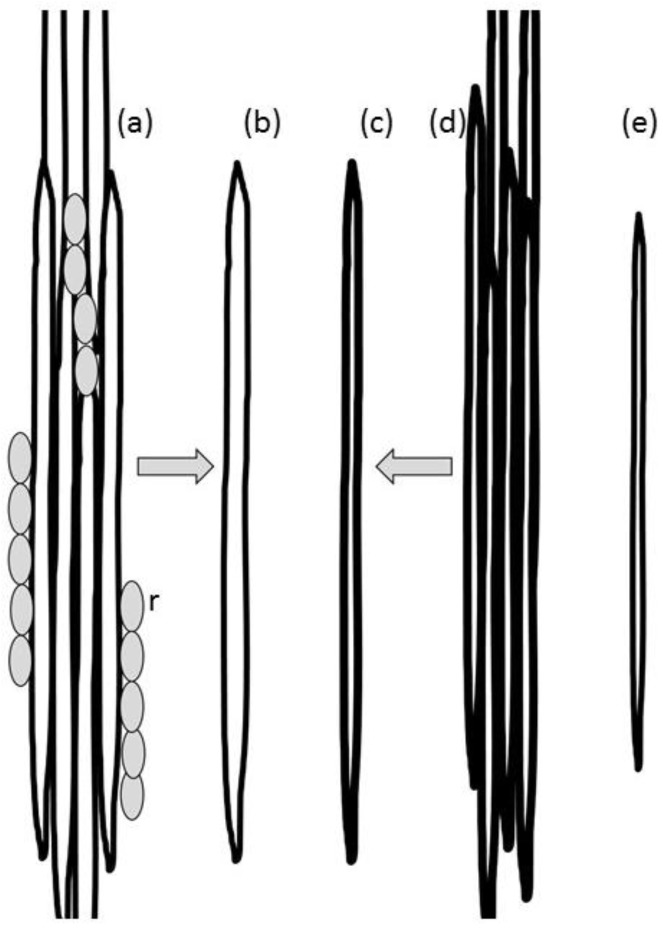
Aspects of various types of fibre. (**a**) Softwood shive, r = ray cell; (**b**) softwood fibre (tracheid); (**c**) bast fibre; (**d**) bast fibre bundle; (**e**) hardwood fibre.

**Figure 3:**
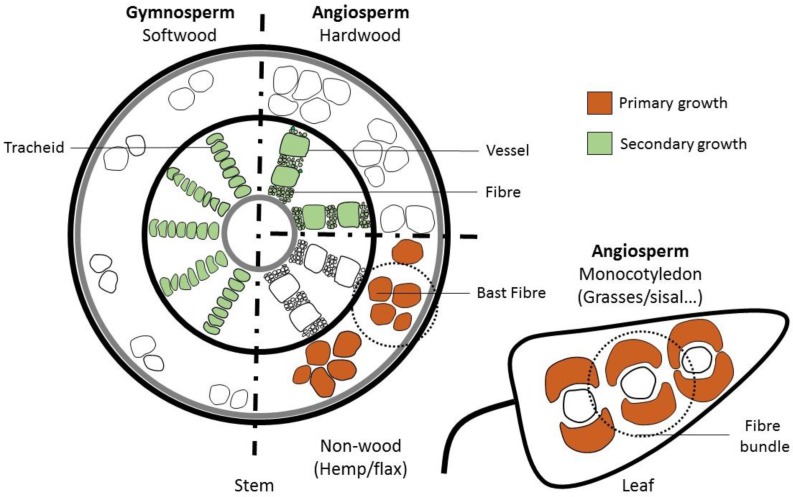
Origins of different types of fibres. The fibres extracted and used are represented in colour. Orange represents the primary growth and green the secondary growth. On the left-hand side, the gymnosperm (softwood) stem contains tracheids. The tracheids have the function of support and sap conduction. On the right-hand side, the angiosperm is divided into two categories. At the top is a hardwood with fibre and vessels. The fibres have mainly a support role, while the vessels are the principal sap conductive elements. The bottom part shows how stem and leaf are grouped in non-wood plants. Bast fibres are found in the stem, and monocotyledon fibres are found in the leaf (excluding bamboo). Both have a support role and are often found in bundles with other elements. The dotted circle represents a bundle of fibres that can be referred as ‘technical fibre’.

‘Fibre’ is a term used by many researchers using natural fibres in composites. However, ‘fibre’ (or ‘fiber’) has a strict botanical meaning, which describes a single elongated, thick-walled, plant cell. The non-botanical definition also applies to the pulp and paper field, and the term ‘shive’ refers to a bundle of fibres (Figure 2a,d). In non-woody plants (hemp/flax), ‘fibre’ may be used to refer to a bundle of non-individualised cells adhering to each other in the same way as they were in the plant. These are also generally known as bast/sisal fibres (Figure 3). In this context, ‘shive’ refers to a bundle contaminated with non-fibrous plant material. For the remainder of this review, the term ‘fibre’ will be used in the context of a single elongated cell [28]. Fibres that remain attached to each other in a long fibrous strand will be referred to as ‘fibre bundles’.

### 3.2. Origin of Fibre

Primary plant growth produces leaves and non-woody material with the function of providing mechanical support and form to the plant [29], while secondary plant growth mainly produces woody material used in both sap conductance and mechanical support [10] (Figure 3). Both types of growth can be used to produce fibres. Primary plant growth produces bast fibres in the phloem (the ‘inner bark’, sometimes called ‘skin’ or ‘bast’) surrounding the stem of certain dicotyledonous plants, such as hemp and flax. Secondary plant growth increases the girth of a stem. Wood is a product of secondary growth, as is bamboo. A comparatively small amount of bast fibre can also be produced by secondary growth. For more details about fibre development, especially initiation, elongation and cell wall formation, see the review of Gorshokova et al. [29]. Overviews of fundamental plant structure can be found in a number of classic texts on plant anatomy (e.g., Esau [30] and Fahn [31]).

### 3.3. Uses of the Terms ‘Primary’ and ‘Secondary’

The terms ‘primary’ and ‘secondary’ are widely used to define different types of plant growth (as outlined above) and also to define different types of cell wall. The commonality between the two is that ‘primary’ mostly refers to elongating and/or enlarging tissues, while ‘secondary’ mostly describes tissue thickening and the development of woodiness. Plant fibres have also been described as being of either primary or secondary origin in terms of their utilisation [32]. Under this definition, ‘primary’ is used to describe plants specifically grown for their fibre (e.g., flax, cotton, wood, etc.), whereas ‘secondary’ is used to describe fibres that might arise as by-products or residues from other industries (e.g., bagasse/sugar cane, sorghum and rice straw). In this review, from now on, the terms ‘primary’ and ‘secondary’ are used to define the cell walls.

### 3.4. Fibre Size

Softwood fibres (botanically called tracheids) can have lengths up to approximately 3.5 mm, diameters of approximately 30 µm and wall thicknesses in the range of 2–3 µm [33]. Hardwood fibres are approximately 0.7–1.5 mm long, with diameters in the range of 13–20 µm and wall thicknesses of 3–6 µm [34,35]. Literature values for the dimensions of bast fibres vary widely. For example, the fibres of kenaf tend to be in the range of 2–3 mm long [36,37,38] (similar to wood), while flax and hemp fibres can be much longer; in the range of 2–5 cm [39,40,41]. Bast fibre diameters vary between approximately 8 and 25 µm (calculated from fibre areas), and wall thicknesses vary between 4 and 12 µm [36,37,38,40]. As mentioned by Morvan [40], some of the reasons for these differences among bast fibre dimensions relate to their ontogeny.

## 4. Cell Wall

### 4.1. Structure and Organisation

**Figure 4:**
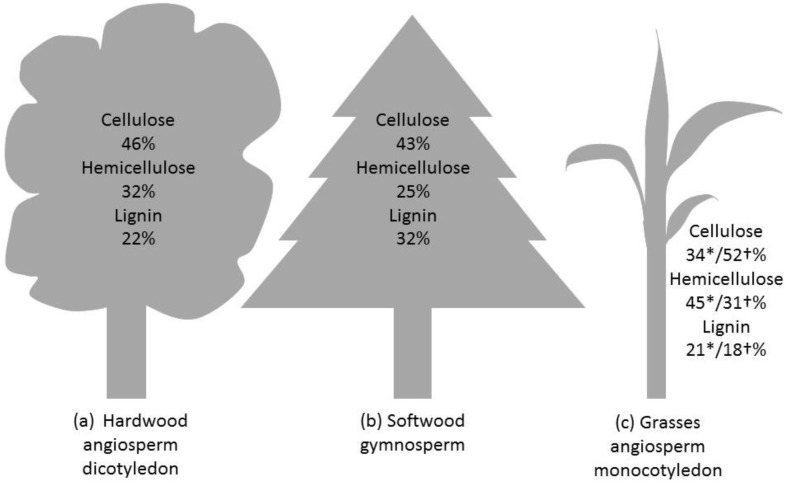
Average composition of stem/straw of lignocellulosic material: (**a**) hardwood; (**b**) softwood; (**c**) monocotyledon (wheat*/corn† straw). Values extracted from Demirbas [42].

**Figure 5:**
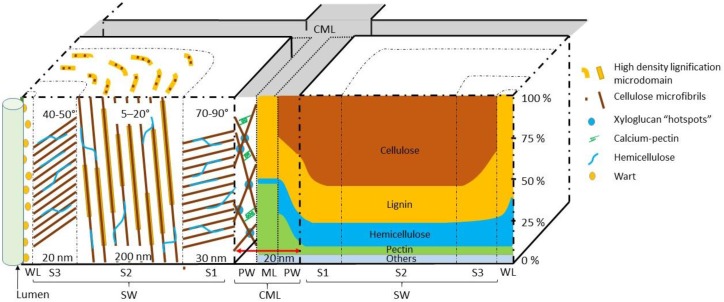
Cell wall model for hardwood from the middle lamellae to the lumen. ML: mid-lamella; PW: primary wall; CML: compound mid lamella; SW: secondary cell wall; WL: warty layer. This scheme represents the cell wall corner between four cells after the secondary cell wall formation. It shows the arrangement and the distribution of the various components of the cell walls. The grey area is the CML separating the four cells. On the left-hand side, the angle of the cellulose microfibrils and the width of the various layers (S1, S2, S3 and CML) can be found. In the secondary cell wall, the hemicellulose is distributed alongside one or linking two cellulose microfibrils. Some microdomains of high density lignification can be found in the S2 layer. In the primary cell wall, the microfibrils are not oriented. The microfibrils are held together in xyloglucan hotspots, and some calcium-pectin complexes have a load-bearing function between different fibres. On the right-hand side is the percent distribution of the various constituents of the cell wall.ML: mid lamellae, PW: primary wall, CML: compound mid lamellae, SW: secondary cell wall, WL: warty layer.

The mechanical properties of the fibres originate in the architecture of the cell wall. The cell wall of land plants is characterized by a framework of polysaccharides, specifically semi-crystalline cellulose microfibrils embedded in an amorphous matrix containing hemicellulose, pectin, polyphenolic material (lignin) and proteins. See Figure 4 for the proportions of cellulose, hemicellulose and lignin in hardwood, softwood and monocotyledon secondary cell wall [42]. The cell wall properties are controlled on many levels, such as by the transcriptional regulation of genes, membrane trafficking and molecular interaction between different components of the cell wall. The wall is organised with three layers: middle lamella, primary cell wall and secondary cell wall (Figure 1) [43]. During cell division (cytokinesis), one cell is divided into two by the formation of the cell plate. This structure will later become the middle lamella. It is a thin layer highly enriched in another type of polysaccharide (pectins). Then, a primary wall is deposited on each side, as the cells expand towards their final dimensions. The primary cell wall is a heavily hydrated gel-like composite comprised mostly of cellulose, hemicelluloses, pectins, structural glycoproteins, some proteins and small quantities of phenolic acids [44,45]. It contains 3–4 layers of cellulose microfibrils with a dispersed orientation. Nevertheless, some degree of microfibril alignment can be observed in tissues where cell elongation is taking place [46]. Middle lamellae and primary cell walls are two hydrophilic contiguous structures ubiquitous in all land plants. Later, after the lignification process, they will become indiscernible and might be grouped under the term compound middle lamella (CML) (Figure 5). Once cell enlargement has finished, the secondary cell walls are formed. The deposition of new material results in the walls becoming thicker. This addition occurs on the inside of the primary cell wall, so this process reduces the internal cell diameter (lumen). Secondary cell walls mainly contain cellulose, hemicellulose and lignin in varying proportions (Figure 4). The secondary cell wall is usually organized into three layers. Adjacent to the compound middle lamellae is an outer layer called the S1, which has transversely-oriented microfibrils with a high angle. The middle, S2, layer makes up the greatest proportion of the wall thickness and has the lowest microfibril angle orientation (angle between microfibril and fibre axis). The inner, S3, layer also has transversely-oriented microfibrils (Figure 5) [47]. The microfibril angle varies depending on the species, maturity, position in the cell wall and growth rate; it dictates some of the characteristics of the plant, such as stiffness. The mechanisms that control changes in cellulose microfibril orientation among wall layers are not known. During the last stage of differentiation, when the lignin is deposited, an additional warty layer that consists of spherical particles (see Section 5.3) with a diameter of 0.1–0.3 µm is created, next to the lumen, on the innermost side (Figure 5) [48]. The mechanical properties of plant fibres depend on the architecture of the secondary cell walls; an understanding of the cell wall architecture is important for not only an understanding of how fibres perform, but how they can be modified to improve their performance.

The overwhelming majority of natural plant fibres used in WPC materials will have secondary cell walls. Cells with secondary cell walls are generally dead at maturity [29]. However, the monocotyledon bamboo has living fibres characterized by a multi-layered secondary cell wall structure. It consists of an alternation of thin layers with high microfibril angles and high lignin content followed by thick layers with low microfibril angles and low lignin content [49]. This type of architecture allows the bamboo to have high mechanical properties.

### 4.2. Function

The middle lamella and primary cell wall are hypothesized to function in cell to cell adhesion, cell expansion and the determination of cell shape [50,51,52]. Primary walls have two conflicting functions: they have to be rigid to withstand the internal and external stresses and, at the same time, have to be pliant to allow cell wall expansion during growth [53]. In contrast, the secondary cell wall structure provides high axial stiffness while at the same time providing high collapse and burst resistance. As it constitutes approximately 80% of the cell wall, the S2 layer has a profound effect on the properties of wood. The characteristics of the S2 layer (such as low microfibril angle, thickness and high cellulose content) are important in generating the wood stiffness (Figure 5). The S1 and S3 layers are relatively thin in comparison, but nevertheless play a critical role in increasing the elastic modulus of the cell in the transverse plane [54,55]. The role of the S1 and S3 layers is to reduce the deformation of the S2 layer under compression and tension. The S1 layer acts as a reinforcing layer preventing excessive radial expansion and rotation of the cell, while the S3 layer helps to avoid sideway collapse when under hydrostatic tension forces. In Booker and Sell’s [56] model, a sandwich structure is composed of the S3, S2, S1, CML, S1, S2, S3 layers (Figure 5). This structure acts as a vibrational dampening system, and the angle of cellulose microfibrils changes the direction of crack propagation during fracture. The fibres will slightly rotate under stress as a result of the microfibril angle arrangement of the S2 layer. This puts the lignin-rich CML under shear stress, and the rubbery nature of the lignin dissipates the stress energy. Overall, the functions of the cell wall are to keep the plant upright, to take up and translocate water and nutrients and to defend from external abiotic stress and biotic attack [57,58]. It is also a system in charge of sensing, processing and responding to internal and external cellular signals [44].

## 5. Molecular Organisation and Interaction of Cell Wall

### 5.1. Cellulose

#### 5.1.1. Cellulose Structure and Synthesis

**Figure 6:**
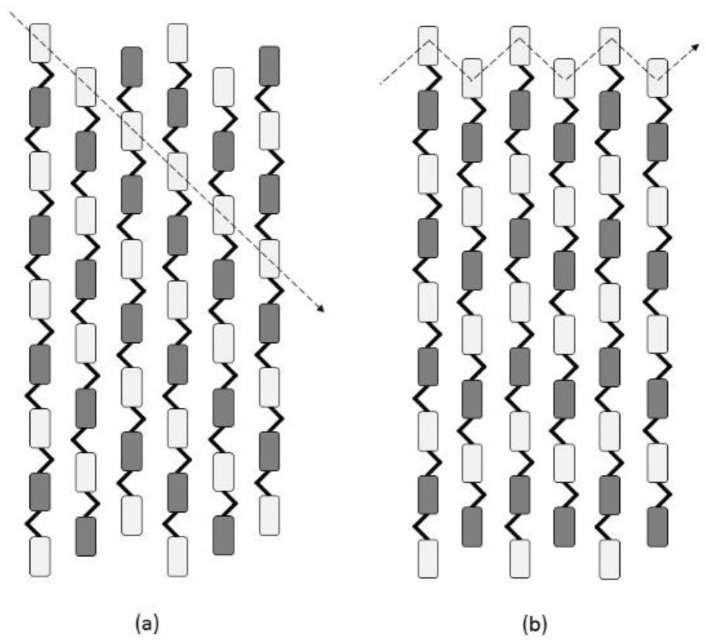
Cellulose conformation. Conformation of: (**a**) cellulose Iα; (**b**) cellulose Iβ. The grey and white rectangles represent glucose molecules linked together by β-(1-4) linkages (**<** and **>**). Two glucose molecules are linked such that each glucose molecule is rotated 180° in relation to each other, represented by grey and white rectangles. These two 180° linked glucose molecules form a cellobiose unit, the repeat unit of cellulose.

Cellulose is a linear chain polysaccharide, consisting of repeating β-(1,4) linked glucose units, with cellobiose (two glucose) being the repeat unit [59]. Each glucose contains three free hydroxyl (-OH) moieties that can interact to form hydrogen bonds. These bonds play a critical role in the aggregation of cellulose chains and determine the crystal structure of the cellulose [60]. These crystalline chains form microfibrils in two arrangements, cellulose Iα and Iβ [61,62] (Figure 6). The thermodynamically more stable cellulose Iβ form is predominant in terrestrial plants [63,64,65].

#### 5.1.2. Cellulose Microfibrils

**Figure 7:**
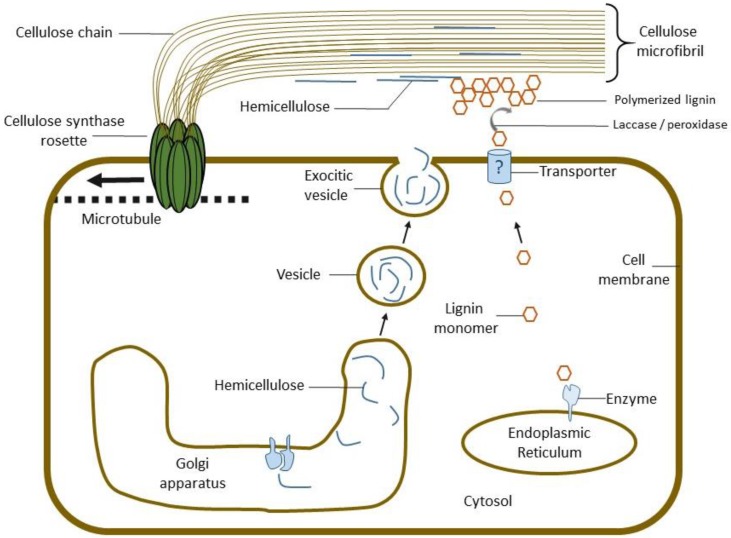
Deposition of cellulose, hemicellulose and lignin in the cell wall. The rosette is a protein complex in the cell membrane producing the cellulose microfibrils. The rosette moves along the microtubules. Hemicelluloses are synthesised in the Golgi apparatus and transported towards the cell wall via vesicles. Hemicelluloses are adsorbed onto the cellulose or other hemicelluloses. Lignin monomers are transported through the cell membrane via an unknown mechanism (probably a membrane transporter). The monomers polymerise together; this process might be due to the actions of laccase and peroxidase enzymes or be a random phenomenon.

Cellulose chain length or degree of polymerisation (number of glucose monomers) is variable (2000–6000) in primary walls and more homogenous (10,000) in secondary walls [66]. The cellulose chains are synthesised in the plasma membrane (the cell membrane) by a rosette-like CesA protein complex consisting of six units that each have six sub-units (Figure 7) [67]. This has led to various hypotheses regarding the number of cellulose chains in microfibrils. The initial ‘bundling’ hypothesis described the presence of 36 cellulose chains, matching the number of CesA proteins per rosette [68,69,70,71]. Such microfibrils would have an hexagonal arrangement [72]. Other researchers have suggested 18 or 24 chains per microfibril [73,74,75,76] or variations in the number of cellulose chains per microfibril [77] with a variety of cross-sectional shapes. A further model of two 18-chain microfibrils ‘twinned’ to give an apparent 36-chain microfibril has been suggested [78]. A microfibril of 18–24 chains should be 2–3 nm wide. Therefore, Anderson et al. suggested that microfibrils aggregate into macrofibrils [79]. This helps explain the observation of a width of 5–10 nm [67,80] and 30–50 nm [81] for typical primary and secondary cell wall macrofibrils, respectively. The aggregation of microfibrils can occur directly between polar surfaces, while aggregation at nonpolar surfaces will likely involve a layer of water molecules between the surfaces [82]. Crystalline cellulose can be sub-divided into a ‘well-ordered’ crystal-surface and ‘poorly-ordered’ crystal-interior [83]. Non-crystalline/amorphous cellulose has been attributed to be a result of mechanical damage or wood pulping treatment rather than being a state of native cellulose [84,85]. One controversial study suggested that cellulose occurs in a non-crystalline state with water molecules randomly distributed on the surface and the interior chains of the cellulose polymer [86]. The use of X-ray and solid state NMR has allowed us to comprehend the cellulose structure, but analyses using these techniques are difficult to undertake due to the inherent overlapping interference of wood components [87]. This highlights the need for on-going work to understand the exact nature and crystallinity of cellulose microfibrils and macrofibrils.

### 5.2. Hemicelluloses

Hemicelluloses are present in the primary and secondary cell wall. They vary widely in structure depending on the tissue and type of plants. Due to their heterogeneity and complexity, hemicelluloses are often treated as a single entity. However, in reality, they include a range of distinct polysaccharides; glucuronoarabinoxylans (GAX), xyloglucan (XG), galactomannan (GalM), galactoglucomannans (GGM), glucomannans (GM), mixed linkage glucans (MLG) and arabinogalactan (AG). Therefore, the hemicelluloses present in the primary and secondary cell wall (angiosperms: monocotyledonous (monocots/grass), dicotyledonous (dicots, e.g., flax, hemp and hardwood) and gymnosperms (softwoods)), and their roles are discussed separately here. The book by Buchanan et al. (2015) provides a full description of hemicelluloses [88].

The model of the secondary cell wall being comprised of cellulose microfibrils within a hemicellulose and lignin matrix [56] generally following the tethered network model [89] persists. Recent studies using NMR, due to the limitation of this technique in the secondary cell wall, have been focused on the primary wall. The latest findings provide new views on the molecular arrangement and interactions of the various components within the primary cell wall. They highlight the importance of the hemicelluloses in the mechanical properties and load bearing of the primary cell wall. We describe the latest results in detail, as similar structures and roles could be extrapolated to the secondary cell wall.

#### 5.2.1. Hemicellulose Synthesis

All of the biosynthetic machinery required to produce the hemicelluloses are localised in the Golgi apparatus (part of the cell’s secretory pathway) (Figure 7). Hemicellulose is then transported in vesicles to the developing cell wall via the plasma membrane [90,91]. The degree of polymerisation is usually estimated at between 60 and 150 residues when in the cell wall [92,93]. Most of the hemicelluloses have high affinity for cellulose, glucomannans having the highest, followed by xyloglucan, xylan and arabinogalactan [94,95,96]. Galactomannans are unable to bind cellulose [88].

#### 5.2.2. Hemicelluloses in the Primary Cell Wall

Primary cell walls provide strength and extensibility to the growing tissues. They can be divided into two broad categories: type I and type II [97,98]. Type I cell walls are found in dicots, some monocots and gymnosperms. They consist of cellulose fibres, neutral xyloglucan (XG), negatively-charged pectin and structural proteins. Type II cell walls are found only in monocots. They are composed of cellulose fibres encased in glucuronoarabinoxylans (GAX) with high levels of hydroxycinnamates and very low levels of pectin and structural proteins. In addition, the cell walls of monocotyledon (e.g., Poaceae) and some related families contain significant quantities of mixed linkage glucans (MLG) [99].

##### Type I Primary Cell Wall

- Composition

**Figure 8:**
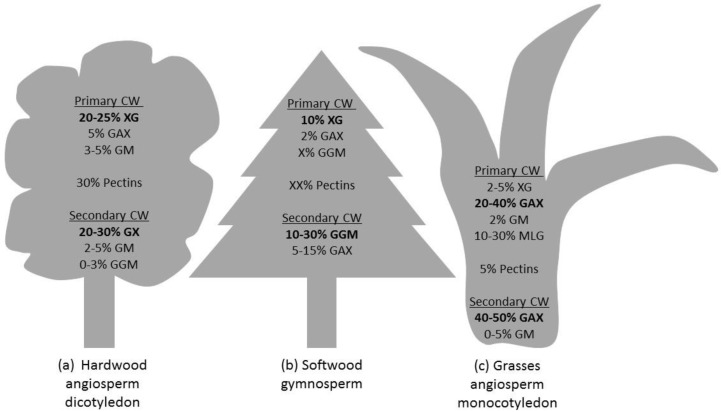
Percentage of hemicellulose and pectins in the primary and secondary cell wall of: (**a**) hardwood; (**b**) softwood; and (**c**) grasses. X represent the presence, but no quantitative data available [90,99,100]. CW: cell wall; GAX: glucuronoarabinoxylans; GGM: galactoglucomannans; GM: glucomannans; GX: glucuronoxylans; MLG: mixed linked glucan; XG: xyloglucan.

Dicot primary cell walls contain up to 20%–25% xyloglucan (XG), 5% glucuronoarabinoxylans (GAX) and 3%–5% (gluco)mannan (GM), as well as over 30% pectin [90]. Angiosperm primary cell walls contain 10% of XG, 2% of GAX, some GGM and pectins (no quantitative data available) (Figure 8) [90,100]. The general structure of xyloglucan is of a β-(1,4)-glucan backbone substituted with xylose residues (75%). Some of these xylosyl residues are substituted with galactosyl or arabinosyl residues, and some of the galactosyl residues are further substituted with fucosyl residues [101,102].

- Structure and Function

Xyloglucan (XG)

The ‘tethered network’ model [89] of the type I primary cell wall, where a network consisting of cellulose-XGs is the main load-bearing component, has been recently revisited. The development of solid state NMR studies has given access to site-specific information regarding the conformation, dynamics, water interaction and intermolecular contacts of the macromolecules in near-native plant cell walls [103,104,105]. For example, solid state NMR studies indicate that despite XG affinity with cellulose [106], only a small proportion of the cellulose surface is actually in contact with the XG [103]. Instead, it is the pectin that interacts with cellulose occupying ~50% of its surface and outcompeting the XG. Upon secretion, a coiled xyloglucan may immediately bind and aggregate to the closest bare cellulose surface it contacts. However, these contact points between XG and cellulose are limited and are called ‘biomechanical hotspots’ [102,105,107,108,109]. The ‘biomechanical hotspots’ could be an intertwined inaccessible amalgam or a monomolecular XG layer between the less hydrophilic surfaces of two cellulose microfibrils. This monolayer would strengthen the cellulose microfibrils’ (CMF) hydrophobic interaction [110]. The presence of XG between two CMFs would reduce the spontaneous direct aggregation/interactions of CMFs with each other [97,102,103,111,112,113]. The absence of XG increases the alignment, interactions and order or crystallinity of CMFs [102,114]. These stiffer and straighter CMFs lead to a greater tension in the cell wall, destabilizing some intracellular components (microtubule cytoskeleton). Moreover, these hotspots might be key target sites for loosening of the cell wall during cell growth [93,109]. Yi and Puri suggested that in the primary cell wall, the stiffness results mainly from CMF and hemicellulose (XG) interactions (75%), while the hemicellulose stiffness contributes to a moderate 24% and the CMF only 1% [115]. Glucuronoxylan is the major hemicellulose of dicot primary cell wall, but a small proportion of GAX is also present. The glucuronoarabinoxylan structure is not yet fully elucidated, and the function is still unclear [92].

##### Type II Primary Cell Wall

- Composition

The type II primary cell walls are the cell walls common among monocotyledons (including bamboo). This form of fibre is commonly used in WPC. The main hemicelluloses are GAX and MLG. The XGs and GM are present in small proportions, 2–5 and 2%, respectively. Only low concentrations of pectins have been detected [99]. GAXs make up 20%–40% of type II primary cell walls. GAX consists of a β-(1,4)-linked xylan backbone with numerous substitutions of (methylated) glucuronic acid ([Me]GlcA) and arabinose residues. The arabinose can be further substituted with ferulic acid. GAX can be highly substituted [90]. MLGs make up 10%–30% of the primary walls of monocotyledons and are unbranched homopolymers of β-linked glucopyranosyl residues, with 70% being (1,4)-linked and 30% being (1,3)-linked (Figure 8) [116].

- Structure and Function

Mixed-Linkage Glucan

MLGs are probably anchored to the cellulose [117], and they form a gel matrix between the CMFs [118,119]. However, the interactions with cellulose are not comparable to either XG or pectin [120] as MLGs do not act as a tether between cellulose microfibril and, therefore, have no load-bearing function. A deficiency in MLG alters cell wall elongation and microfibril orientation (possibly shorter and less organized CMF). This suggests that MLG may have a role in maintaining proper cellulose deposition and organisation [121].

- Relevance of Primary Cell Wall Model

From the fibre perspective, the primary cell wall encases the secondary cell wall. Recent discoveries are shifting the way we think of the role (load bearing and structural arrangement) of the various elements in the primary cell wall. Solid state NMR has changed our vision of the primary cell wall, by giving us insight on interactions in the native primary cell wall, but this approach is more challenging in secondary cell walls, and the way the various components are interacting is less understood. Thus, current information on the secondary cell wall structure has been obtained using indirect methods.

#### 5.2.3. Hemicelluloses in Secondary Cell Walls

Secondary walls determine the shape and function of cells, as well as their response to the environment [122]. Like primary cell walls, secondary cell walls also consist of polysaccharides, cellulose microfibrils and various hemicelluloses, but the water content is lower due to the presence of the hydrophobic polyphenol lignin.

##### Composition

In monocotyledons, the composition of secondary cell walls is relatively similar to the primary cell wall, but the proportion of GAX is higher (40%–50%), while the amounts of MLG, XG and GM are low [90]. Recent studies report that MLG may also be substantially present in specific tissues of some monocotyledons (Figure 8) [123,124]. In dicotyledonous plants, the major hemicellulose is glucuronoxylan (GX) (20%–30%) along with small proportions of mannans and glucomannans (GM) (2%–5%) [99]. In softwoods (e.g., conifers), the main hemicellulose of the secondary cell wall of fibres (tracheids) is galactoglucomannans (GGM) (10%–30%) [125,126], and 5%–15% of GAX is also present.

Glucuronoxylans consist of a β-(1,4)-linked-d-xylopyranosyl backbone with substitutions of α-(1,2) glucuronic acid and 4-*O*-methyl glucuronic acid units, collectively termed [Me]GlcA [90]. Mannans have a β-(1,4)-mannose backbone, but can be substituted with galactose to produce galactomannan (GalM). In contrast, glucomannans have a mixed backbone of β-(1,4)-mannose and β-(1,4)-glucose residues in a non-repeating manner. These can have galactosyl side chains to form galactoglucomannans (GGM) (Figure 8) [127,128,129].

##### Structure and Functions

- Xylans

The GAX contain three domains; the first one is attached onto the cellulose; a linker makes the transition to the third one, which promotes the separation of CMFs. The middle domain could be coupled to the gel-like MLG [119]. The arabinose residues in GAX are esterified with the phenolic compound ferulic acid. Feruloyl esters can undergo oxidative coupling with other phenylpropanoids in the vicinity. Therefore, a GAX molecule that is adsorbed on a cellulose MF can directly interact with lignin via the ferulic acid present on its arabinose side chain. It can also potentially do this with other GAXs via a di-ferulic acid cross-linking [130,131,132]. This structure is key for the cell wall strength of monocotyledons [133,134].

**Figure 9:**
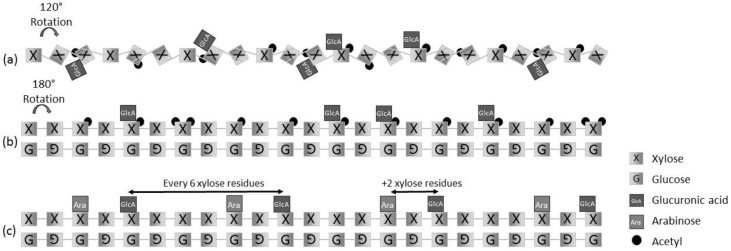
Xylan substitutions in hardwood and softwood. (**a**) Hardwood unevenly distributed substituents domain; every xylose has a rotation of 120° (three-fold screw). As the substitutions are uneven, the xylan cannot bind the cellulose chain made of glucose. (**b**) Hardwood evenly spaced substituents domain; every xylose has a rotation of 180° and all decorations face one side allowing the adsorption onto the cellulose. Unevenly and evenly distributed domains are probably on one xylan molecule of around 120 xylose residues. (**c**) Softwood xylan decoration; the glucuronic acids are every six xylose residues, and the arabinose are at plus two xylose residues from the glucuronic acid. This domain xylan molecule can be adsorbed onto the cellulose.

In dicots (hardwood), the xylose residues forming the GX backbone are substituted with *O*-acetyl and glucuronic acid groups (55% and 10%, respectively) [135,136]. A precise pattern has been detected for both types of substitution [137,138]. The distribution pattern of the glucuronic acid substituents shows two distinct domains. The first domain contains predominantly evenly-spaced substituents (average space of eight residues between GlcA substitutions). The two-fold helical screw (180° twist) configuration of the backbone produces a conformation that enables it to fit into the grooves of the most hydrophilic surface of a rectangular microfibril (Figure 9). This is possible as the side chain residues all face away from the cellulose microfibrils. The second domain may occur in the same molecule, but these substitutes are tightly clustered and do not present a preferential even distribution. This impairs the association of this domain with the CMF, leading to a relaxed two-fold (120° twist) of its backbone [139,140]. It has been suggested that this arrangement produces a non-adsorbing portion between two even domains adsorbed onto CMFs and/or could promote interactions with lignin (Figure 9) [137,138]. Acetylation of xylose units and methylation of some GlcA units, by reducing the hydrophilicity, may enhance xylan cross-linking to the lignin and reduce its accessibility [141,142]. In softwoods, the recently characterised pattern of substitution is different. Acetylation is absent; nevertheless, GlcA are arranged every six xylose residues, and the arabinose is at plus two of the GlcA (Figure 9) [142]. Various potential arrangements of the cellulose-xylan composite have been tested using a molecular dynamic simulation under a shear load [143]. However, the biological significance of cellulose-xylan-lignin interactions are still to be fully determined [144].

Xylans (especially those with low levels of substitution) have been shown to be localized at the interface between S1/S2 and in the S3 layers. These areas show changes in the CMF orientation, from a disordered to a transverse, then more horizontal orientation [46,129]. Therefore, it has been hypothesised that xylans may function as twisting agents controlling the orientation, aggregation and alignment of microfibrils. In *Pinus radiata*, for example, highly-substituted xylans have been found across all areas of the secondary, but not primary cell wall [129,145,146]. Due to their frequent branches, xylans may function as a link between rhamnogalacturonan-I (RG-I) polymers and cellulose microfibrils [147]. The enzyme xylan endotransglycosylase releases mechanical stress arising during secondary cell wall deposition, affecting the cellulose microfibril angle (MFA) [148]. This enzyme probably acts by detaching the xylan from the CMF.

#### 5.2.4. Other Secondary Cell Wall Hemicelluloses

Hemicelluloses are deposited prior to or concurrently with lignin, so it has been hypothesised that they could be part of the process controlling lignification [149,150,151]. Mannans may associate with xylans and lignin [152,153] and may have a strong association with cellulose [95,96]. Mannans and xylans are mainly deposited at the S1/S2 boundary prior to lignification [146,154,155]. These polysaccharides may act jointly as a barrier to the infiltration of monolignols into the middle lamella, reducing the lignification of the outer wall. In contrast, another hemicellulose, β-(1,4)-galactan, is only associated with areas of increased lignification [156,157].

The use of super-resolution techniques (scanning near-field optical microscopy (SNOM)) revealed the universal presence of circumferential microdomain patterns of cellulose and lignin polymerisation [158] that had been suggested previously from the observations of radial agglomerations [159,160,161,162,163]. Hemicellulose deposition on cellulose MF could explain these secondary cell wall arrangements.

#### 5.2.5. Hemicellulose Conclusion

This overview of the hemicelluloses shows that they have a wide variety of properties. Even though the role of hemicellulose and the molecular architecture of the cell wall is not yet clear, all of the available data tend to suggest a key involvement of non-cellulosic polysaccharides as regulators/facilitators of cell wall assembly. Hemicelluloses affect cellulose orientation; they regulate the tensions between primary and secondary cell walls; strengthen the interface between cellulose and lignin; and might even influence lignification processes.

### 5.3. Pectins: Composition Structure and Function

Pectins are complex polysaccharides, but are not classified amongst hemicelluloses. Pectins are negatively-charged acidic polysaccharides rich in galacturonic acid (GalA) residues. There are three types of pectin in dicot primary walls: homogalacturonan (HG), rhamnogalacturonan-I (RG-I) and rhamnogalacturonan-II (RG-II). Homogalacturonan is composed of unsubstituted α-(1,4)-linked galacturonic acid (GalA) (~200 units). Rhamnogalacturonan-I possesses a backbone of alternating rhamnose and GalA subunits that are decorated with galactan, arabinan and arabinogalactan side chains of varying lengths. Rhamnogalacturonan-II is more complex, as its linear GalA backbone can be modified with four different side chains and includes twelve different monosaccharide constituents [164]. Moreover, side chain A contains apiose residues that can form borate diester linkages to crosslink RG-II molecules, generating RG-II dimers [165,166]. In general, pectin in plant cell walls consists of 50%–70% HG, 20%–30% RG-I and 10% RG-II [167], with substantial cross-linking between the structures [168,169,170].

Pectins (most likely RG-I) are detected close to the cellulose surface (~1 nm), but molecular crowding is not the reason for the cellulose-pectin spatial contact. The cellulose-pectin spatial proximity is an intrinsic feature of the never-dried primary cell wall, and some of the pectins may even be entrapped within or between cellulose microfibrils [171]. Dehydration does not cause irreversible changes, as lyophilization followed by rehydration restores the wall polysaccharides to their original structure and dynamics [172]. Therefore, pectins serve as mechanical tethers between microfibrils. Consequently, as for the cellulose-XG cross-links, Ca^2+^-pectate cross-links also play a major load-bearing role and may also be a key regulator of cell wall adhesion [50,173]. Pectins attract and bind water, which reduces the water mobility in the cell wall [172]. Cross-linked RG-II can influence the strength and porosity of the primary cell wall [165,174]. Moreover, some pectins may be covalently linked to arabinogalactan proteins [175] or XG, but the prevalence and structural significance of such hybrid molecules remain to be determined [176,177]. The primary wall of higher plants has been viewed as a single cohesive network of polysaccharides [73,171]. It has been suggested that pectins have a role in controlling the shape of lignin molecules in the CML [152].

### 5.4. Lignin

After cellulose, once it is deposited, lignin is the second most abundant polymeric organic substance in plant cell walls. Lignin is a complex three-dimensional aromatic molecule composed of phenyl groups. Lignin acts as a cement and has a supportive structural function, it also protects the hydrophilic cellulose and hemicelluloses [178]. Lignin is synthesized by the coupling of hydroxycinnamyl subunits called monolignols; mainly coniferyl, sinapyl and *p*-coumaryl alcohols. The most abundant monolignol in hardwoods is sinapyl alcohol, and this leads to syringyl S-units in the lignin polymer. In softwood coniferyl alcohol is the main monomer leading to guaiacyl G-units. *p*-coumaryl alcohol or H-units can also be found [179,180].

The biosynthesis of monolignols is well described [181]. Once synthesised inside the cell monolignols are transported to the developing cell wall through the cell membrane. The following polymerisation process, is less clear [182]. It results in a radical coupling creating a seemingly randomly branched plant biopolymer. This process might involve enzymatic reactions of laccases and peroxidase. Lignin is deposited within the polysaccharide cell wall framework by infilling voids [183].

Lignification constitutes the last stage of the cell formation and is part of a process of programmed cell death. In woody tissues, lignification likely occurs in parallel with polysaccharide deposition [184,185], but there is evidence that, in some cases, it happens post-mortem, with neighbouring cells providing both lignin monomers (monolignols or dilignols) and reactive oxygen species (ROS) to dead lignifying tissues [182,186,187]. After lignification, the distinctions between the two primary cell walls surrounding the middle lamella are lost and are then referred to as the compound middle lamella (CML). The CML can comprise 10%–12% of the woody tissue volume in softwood [188].

Polymerisation starts in the highly lignified cell corner, then continues in the CML and secondary wall (S1, S2 and S3), resulting in a trend of lignin content from high to low in these layers [189,190]. The warty layer between the S3 and the plasma membrane is composed of highly cross-linked lignin precursors that are formed while the cell is in the final stage of lignification and death [191]. CML has a 50%–70% lignin concentration (*w*/*w*), while the S2 region contains about 20% [190,192]. Although the S2 layer in normal wood is typically uniformly lignified, it may occasionally show concentric variations in lignification [158]. In the CML, lignin is structurally differently from the secondary cell wall lignin; more *p*-hydroxyphenylpropane and condensed bonds are found (mainly C–C bonds (aryl ether)), whereas non-condensed (*O*-4 aryl-alkyl ) bonds in the S1 are dominant [193,194]. There are van der Waal interactions between lignin and cellulose microfibrils via hydrogen bonds. This results in a cohesiveness between the lignin/hemicellulose matrix and the crystalline cellulose. The weakest linkages are between the less crystalline cellulose regions and the lignin/hemicellulose matrix [195]. A high proportion of lignin is in contact with these polysaccharides [152,196], but the full nature of the bonding with the hemicelluloses has yet to be determined. However, covalent bounds are only present between lignin and hemicelluloses [197]. Three types of linkage are commonly acknowledged to occur in wood: phenyl glycosides, esters and benzyl ethers [198]. These linkages can be strong and resilient; the difficulty in breaking them limits chemical pulping process efficiency [197]. In softwoods, the high degree of lignin condensation contributes to the difficulty of its breakdown and processing.

Lignin acts as a gluing matrix for cellulose microfibrils. It also stabilizes cell size by increasing the rigidity of cell walls and enhances the compression strength and support of the cell, as well as adhering adjacent cells to each other. Lignin also affects the water permeability of the cell wall (for sap conduction) and helps protect against pathogens [199].

### 5.5. Water

**Figure 10:**
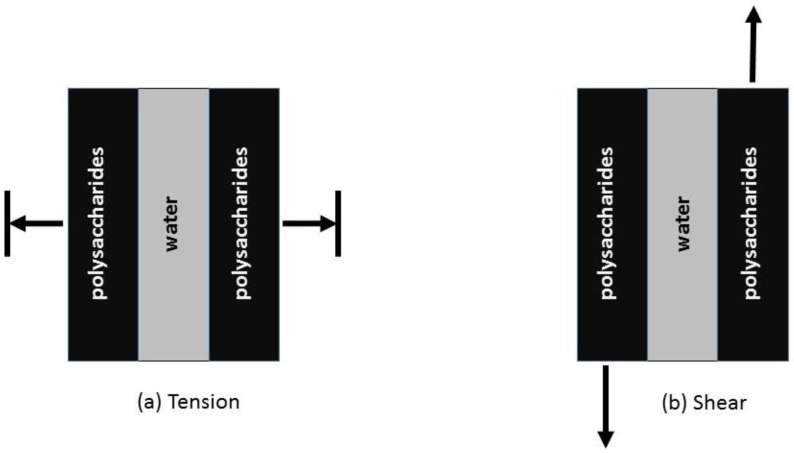
Properties of water in the cell wall. (**a**) A water layer impairs the separation of two polysaccharide elements through surface tension; (**b**) but promotes sliding when the polysaccharides are under shear pressure.

**Figure 11:**
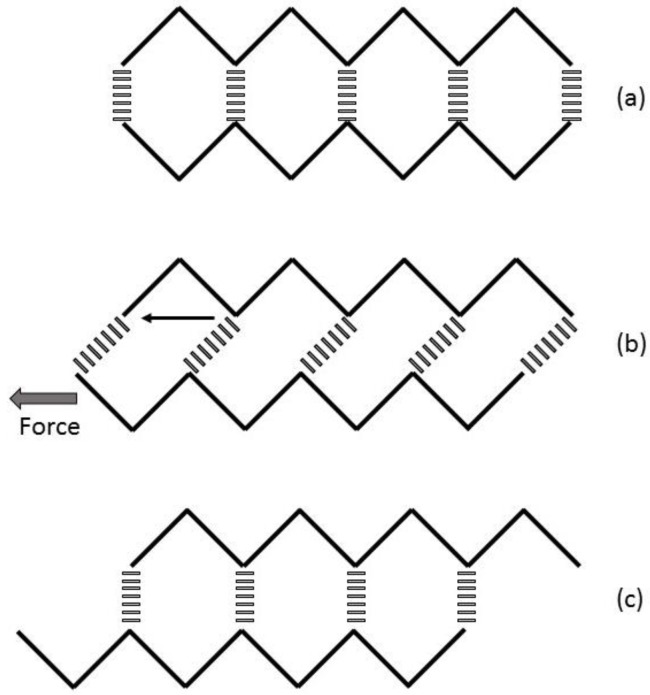
Adaptation of the ‘rip, slip and stick’ model of molecular translation in a lignocellulosic material. (**a**) On the initial hydrogen bonding conformation; (**b**) a force is applied causing the hydrogen bonded network to stretch; (**c**) then after the applied force has ceased, the hydrogen-bonding network reforms in the (new) most favourable position.

Wood fibres are a viscoelastic material; their mechanical properties are affected by temperature, moisture and load [200,201,202]. Two kinds of water are present in living wood: free water (predominantly in the cell lumens) and bound water (inside the cell walls). All of the bound water molecules are directly bound to one or two sorption sites [203], mainly hydroxyl groups [204]. Most of the sorption sites are found in the hemicelluloses followed by cellulose and lignin [205]. Only a third of the hydroxyl groups that are on the surfaces of the cellulose microfibrils are available as sorption sites. The other two thirds of hydroxyl groups are involved in the interaction between and within cellulose chains [206,207]. Due to the acidic pH of wood (pH 4–6) [208,209], there is an 80-fold increase of oxonium (H_3_O^+^) ions compared to a neutral solution (at 25 °C) [210]. Their presence increases the water layer organisation, lubricates the interfaces and softens the lignin/hemicellulose matrix [143]. Under ambient temperature conditions, when the moisture content rises above a relative humidity of 70%, the state of the hemicelluloses is above its glass transition temperature, which leads to it softening. This state allows the hemicelluloses to accommodate even more bound water and has the greatest effect on cell wall swelling [204]. The layer of water between two polysaccharide structures impairs their separation due to the surface tension, but improves their sliding under shear (Figure 10). Hydration is an important factor for the molecular recovery mechanism that occurs after an irreversible deformation. This ‘rip-slip-stick’ self-repair mechanism is likened to Velcro^®^ [211,212]. Initially, there is a ‘tearing’ of the hydrogen bonds (the ‘rip’) in response to an applied force. This is followed by a translation of the polymer chains relative to each other (the ‘slip’), and finally, a reattachment of the hydrogen bonding network (the ‘stick’) (Figure 11). This mechanism explains why wet wood is the only material able to exceed its plastic limit without damage. When stretched beyond this point, wet wood shows irreversible deformations and, yet, does not flow as plastics do [211]. As soon as the excess stress is released, the original stiffness is restored. In other words, wet wood shows permanent plastic deformation without significant mechanical damage due to its self-healing properties [213,214].

The strength of hydrogen bonds between a hydroxyl group and a linked oxygen is in the order of 12–25 kJ·mol^−1^, while the covalent bonding of oxygen to hydrogen is 460 kJ·mol^−1^. Therefore, the sum of an array of hydrogen bonds (between 18 and 38) is stronger than one covalent bond [215]. When fibre is dried and the layer of water is removed, the water is no longer mediating a dynamic exchange of hydrogen bonds between cellulose and hemicelluloses [216]. The hydrogen bonding becomes more static, impairing the rip and slip part of the Velcro^®^ effect. Therefore, the fibre is less flexible and more prone to failure. To demonstrate this mechanism, ammonia (which has the potential to break hydrogen bonds) has been added to dry wood, and its presence has been shown to increase fibre flexibility [217,218]. Moreover, dehydration leads to a decrease in the spacing between the cellulose hydrogen-bonded sheets of chains. This process, often referred to as hornification, induces deformations as it impacts the cellulose shape, chain angle, compaction of its crystal structure and intra-chain bond formation [213,219]. Such changes are partially reversible with rehydration and, in part, may account for the observed drying hysteresis in the re-watering of wood.

## 6. Fibre Extraction Process and Related Physical Properties

### 6.1. Wood Pulping Process

Individual fibres are usually extracted from wood by either chemical or mechanical methods [220]. These two processes have considerably different yields and produce fibres with different inherent properties. In general, chemically-produced fibres are longer, less damaged, more flexible and more hydrophilic than mechanical pulps.

#### 6.1.1. Chemical

**Figure 12:**
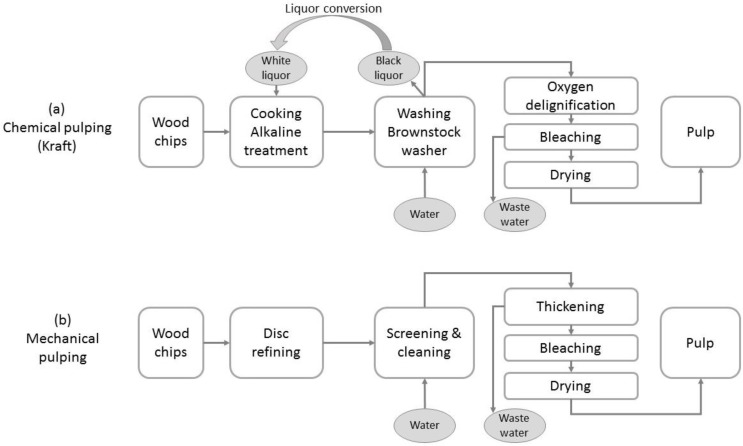
Stages of the pulping process: (**a**) chemical pulping; (**b**) mechanical pulping.

The alkaline Kraft process [221] is the predominant chemical extraction method where the fibres are separated from each other by dissolving the lignin-rich middle lamellae (Figure 12a). Hemicelluloses are also removed during this process, and the lignin and cellulose are decoupled by oxidation and hydrolysis. As a result, the stiffening effect of the lignin is lost [222]. After chemical pulping, the fibre surface may still be enriched in lignin, even though this may represent only a small amount of the original lignin quantity [223,224,225]. This lignin originates from the dissolved lignin present in the pulping liquor being re-adsorbed on the fibre surfaces [226,227]. In addition, extractives and metals might precipitate on the fibre surface during the subsequent acidic bleaching process [228]. Kraft fibres are considered to be highly hydrophilic. Kraft pulping has a yield around 50% and is energy neutral, as the dissolved lignin is burnt to power the process. This chemical process is expensive, however, partially due to the troublesome waste management [229]. Kraft pulp fibres have higher strength [230] and flexibility, meaning they have a higher commodity value than mechanical pulp. It is important to note that chemical pulps are often subsequently mechanically refined to increase fibre flexibility and to disrupt the cellulose microfibrils in the outer wall layers.

#### 6.1.2. Mechanical

Mechanical pulping is a high yield (90%–98%), energy-intensive (1000 kWh/tonne [231]), fibre separation process. It requires an attrition device, such as a refiner, to mechanically separate the fibres from each other using shear forces (Figure 12b). The attrition can cause fibre damage, however. The extent of the damage can be partially controlled by altering the wood elasticity prior to attrition (steam and/or chemicals). The predominant mechanical pulping processes currently in use are: thermomechanical pulping (TMP), chemi-thermomechanical pulping (CTMP) and high temperature thermomechanical pulping, more commonly referred to as the medium density fibreboard (MDF) process.

For the TMP and CTMP processes, fibre separation is principally achieved at low temperature (100–140 °C) by physically grinding and rupturing the S1/S2 interface [232] to produce relatively hydrophilic fibres. These fibres still contain secondary layers (microfibrillar structures) and a few patches of remaining middle lamella. This heterogeneity confirms that physical rupture occurs through various structural layers [233]. The presence of lignin-rich hydrophobic middle lamellae remnants reduces inter-fibre bonding [234]. In contrast, the high temperature (165–180 °C) MDF process causes fibre separation within the middle lamella and, thus, results in hydrophobic fibres with a smooth lignin film coating. In general, mechanical pulp fibres are stiffer and have lower collapsibility than chemical pulp fibres, as they retain substantially higher levels of lignin.

Another important aspect regarding the behaviour of the fibres is the presence of hemicelluloses on the surface that can influence strength, bonding potential and hornification properties [235]. These can be selectively removed by chemical or enzymatic treatments.

#### 6.1.3. Annual Crop Pulping Process

For hemp and flax, the bast fibre can be extracted by retting. The retting process weakens the middle lamella by the action of microorganisms or enzymes. The retting of the fibres is followed by breaking, scutching and hackling. Breaking is performed by passing the stems between rollers; scutching separates the bast fibre bundle from the xylem; finally, hackling thins the fibre bundles by passing them through a series of combs. Decortication is done by hammer milling or roller milling. Hammer milling is a high throughput method, but creates small and damaged fibre bundles (1–10 cm). Roller milling is the opposite, as it can produce long undamaged fibre bundles at a slow rate (30–60 cm) [236].

### 6.2. Fibre Damage

Extracted natural fibres always show some level of disruption or damage [237]. These are often called dislocations or microcompressions in wood fibres [238] and kink bands in bast fibres. Deformation and damage of natural fibres starts within the plant as the plant is subjected to forces, such as wind and weight bearing [56,237]. Damage is exacerbated by fibre extraction and other processing [237,238], and the severity of the damage depends on the nature and severity of the treatment [239]. Extrusion and injection moulding processes further damage fibres, reducing fibre length and decreasing mechanical properties. The flexibility of natural fibres means that breakage generally happens after they have gone through repeated deformation cycles [240].

Deformations will preferentially occur in regions of the cell with a low density of elastic components, i.e., where hemicellulose and lignin are depleted. Detailed descriptions of fibre damage can be found in a number of reviews [237,238,239]. Dislocations represent localised buckling of the cell wall as in a compression failure (a micro-compression) [238] and can also show signs of wall delamination [241,242]. They are generally visualised using cross-polarised illumination where the localised change in cellulose orientation results in a change in extinction angle and associated interference colour.

Relationships have been suggested between the strength of wood fibres and their lengths after injection moulding [243], and the presence of dislocations has been shown to correlate with reductions in fibre stiffness [244] and strength [245] for wood and non-wood fibres [246,247,248]. However, as discussed in a review by Hughes [240], whether or not dislocations cause a reduction in fibre strength is less clear, and levels of induced fibre damage in wood pulp were shown to have a minimal effect on WPC properties [249]. This is because dislocations are due to local mechanical distortions of cellulose fibrils and may play only a minor role in strength reduction [250]. Thygesen et al. [242] have shown that dislocations can be removed, or straightened out, under tensile stress with no measurable effect on fibre stiffness. They suggest that the presence of dislocations indicates that the fibre has experienced mechanical stresses that have weakened the fibre and do not necessarily act as the site of failure. Direct studies of fibre failure are limited, but Le Duc et al. [240] have shown that flax fibres tend to break at kink bands under shear conditions, whereas in wood fibres, failure under tensile force generally initiates at existing cracks in the fibre wall, rather than dislocations [251]. Wood polymer composite processing may induce greater levels of damage in non-wood fibres than in wood fibres, which may be related to differences in fibre flexibility and geometry [252].

### 6.3. Fibre Mechanical Properties

Plant fibres vary in their mechanical properties. This is because fibre morphologies vary for a large range of reasons: woody plants and non-woody plants have fibres that perform different functions; they vary in type and composition. Between taxonomic classes of plants there are differences in fibre anatomy, often distinctive enough to identify their origin to a given species [39]. Within a species, fibre morphologies vary due to climate [253]. Within a single tree, morphologies often vary due to season (e.g., growth rings in wood). At the same point in time, fibres growing in different positions in a plant will have different morphologies [254]. The range of mechanical properties of natural fibres and their WPCs have been summarised by a couple of authors [2,32]. 

## 7. Plastic-Reinforced Fibre

### 7.1. Extrusion

Wood particles have long been used as fillers for extruded WPCs. When wood particles, or wood flour (e.g., derived from shavings or sawdust), are used in WPCs there is often an increase in WPC stiffness, but little improvement in strength [255,256]. A major reason for this is that the wood particles/flour consist of a number of agglomerated wood fibres that have never been separated out into their component fibres. As such, the particles are often large, but with modest aspect ratios (the proportional relationship between its width and its height) and, therefore, limited reinforcing potential. Fibres that have been released from the wood by pulping have greater aspect ratios. A number of researchers have investigated using pulp fibres for WPC reinforcement after recognising that pulp fibres provide greater reinforcing potential than wood particles [257,258,259,260,261,262,263,264].

There are two main problems with using wood pulps in WPC materials. Firstly, loose pulp can be difficult to handle and process. Fibre entanglement can cause blockages (bridging) during feeding into the extruder [265] and result in a variable production rate and fibre loading levels. Moreover, pulp dust is an explosion hazard [266]. The second problem is that pulp fibres generally require drying before extrusion. One option to solve the feeding issues above is to produce wood pellets from dry wood pulp. Often, this is achieved by pulp drying, which is similar to the papermaking process. Although this solves the feeding issues, it does generate another series of processing challenges, in particular how to achieve good dispersion of the wood fibres during extrusion and, thus, throughout the WPC.

### 7.2. Dispersion

#### 7.2.1. Reprocessing

To reach the full potential of a WPC, a homogeneous dispersion of the fibres is required. Generally, compatibilisers are added during WPC processing and this improve the dispersion of pulp fibres [260,263,267] and wood flour [268,269,270]. Multiple cycles of reprocessing (extrusion or injection moulding) lead to an increased dispersion, but at the same time, reduce fibre length. As fibre length is reduced, the aspect ratio is also reduced, which usually leads to a decrease in mechanical properties [271,272,273,274]. However, with bast fibre bundles, the shear forces in the extruder/injection moulder further facilitate the individualisation of fibres, reducing the fibre bundle length, but also its diameter, leading to an increase in aspect ratio [275,276]. A pectinase treatment prior to WPC processing [277] for improved retting also enhances bast fibre bundle individualisation. In the case of the wood flour, it is unlikely that the dispersal of the wood particles is accompanied by a dissection of the particles into individual fibres [263,278].

#### 7.2.2. Pelletisation

To improve the high-throughput feeding of fibres during the extrusion process, pulp pelletisation has been investigated. The production process of pellets is similar to paper making. As the water is removed from the pulpsurface tension forces bring fibres together via fibre-to-fibre hydrogen bonds [279,280]. Literature values for the strength of fibre-to-fibre bonds lie roughly in the range of 2–7 MPa [280,281]. This is considerably weaker than the measured ultimate tensile strength of wood fibres (300–1400 MPa) [282,283], but the fibre-to-fibre bonds need to be broken again for the dispersal of the fibres to be effective. In paper recycling, this is a relatively simple process, as the reintroduction of water breaks the fibre-to-fibre hydrogen bonds. The dispersal is improved as the level of re-wetting increases [284]. However, the application of shear forces is also generally necessary to completely break up each group of fibres [284]. Breaking up pellets of bonded pulp fibres is more difficult in a non-aqueous environment. It is also important to note in this context that the strength of bonded pulp fibres is governed by more than just the strength of the fibre-to-fibre bonds. According to Niskanen and Karenlampi [285], the tensile strength is governed by: the breaking stress of the fibre bonds, fibre strength, length and width and the relative bonded area between fibres. More details on fibre bonding can be found in Niskanen et al. [286]. This means that considerable thought needs to go into optimising pulp pellets for WPC processing. In particular, the drying and the density of pulp sheets are critical in order to limit bonding, fibre entanglement and to enhance fibre dispersal.

Bengtsson et al. [257] and Le Baillif and Oksman [258] pelletised softwood chemical pulps, but concluded that there was significant fibre breakage during either the pelletisation process or subsequent processing. Looking at a range of pulp pellets compounded in polylactic acid (PLA) and polypropylene (PP), Peltola et al. [264] found that there was better fibre dispersal in PLA than in PP. This can be attributed to the higher melt viscosity of the PLA resulting in higher shear forces to help break up the pellets and fibres [264,287]. Even so, there was also a reduction in fibre length after PLA processing. Bengtsson et al. [257] suggested that the fibre length reduction and fibre damage experienced during pelletisation and processing resulted in only a marginal reinforcement gain compared to wood flour. Looking to improve fibre dispersal and handling, Le Baillif and Echtermeyer [259] blended bleached sulphite fibres with carboxymethyl cellulose (CMC). The addition of CMC in the pelletisation process helped to preserve some of the fibre length during pellet production. On the other hand, the CMC made the pellets stiffer, impeding gravimetric feeding, and the increase in fibre-to-fibre bonding made the dispersion worse during extrusion. Warnes et al. [265] used the MDF process and a thermoplastic binding agent to produce fibre-rich pellets that were both easy to feed into an extruder and readily dispersed the fibres during compounding. This is commercially available as WoodForce™.

## 8. Fibre Improvement

### 8.1. Post Pulping Improvements

Natural fibres can reinforce various polymeric matrices as a result of their good strength and stiffness. However, their high hydrophilicity results in moisture absorption [6] and poor compatibility with hydrophobic matrices [288]. The abundant hydroxyl groups present on cell walls are responsible for their hydrophilic characteristics. Attempts have been made to modify these groups in order to improve durability, dimensional stability and compatibility with the matrix. Chemical methods are commonly used to make the fibres more compatible with the matrix [289]. The fibres can be modified (sized) before extrusion or during the polymerisation of the matrix. The most common chemical treatments of fibres, improving the WPC performance, involve silane [290,291], maleic anhydride [292,293,294,295], mercerization [296,297,298] and acetylation [9,299,300]. To make the fibre more hydrophobic, several other types of treatment, such as permanganate [301], acrylation [302], isocyanate [303], benzoylation, fatty acid derivation (oleoyl chloride), enzymatic [235,304], fluorination [305], octadecylamine [306], PEI (polyethylene imine), CaCl_2_ and Ca(OH)_2_ have also been investigated. Some physical methods involving treatment by plasma [307], heat, corona, laser or γ-ray [308,309] have also been used and impact positively on the mechanical properties, such as stiffness, of the WPC. Additionally, nano-cellulose has been used with non-wood fibre to create ‘hairy’ or ‘fuzzy’ fibres with a nano-structured hierarchical interface [310,311,312,313,314]. Such approaches have been shown to increase the tensile stiffness of the resulting WPC by 40% [311].

### 8.2. Pre-Pulping Improvements

Another strategy aimed at improving fibre quality is through conventional and science-based plant breeding [315]. The conventional techniques include: mass selection; cross-breeding; in-breeding; and hybrid breeding [316]. Biotechnology tools can assist and accelerate traditional breeding. The use of high-throughput marker-assisted selection using quantitative trait loci (QTL) [317] combined with the chromosome segment introgression line (CSIL) [318] has been revealed to be a powerful combination. For example, the strategy of breeding plants with rare defective alleles (BRDA) allows a reverse genetics approach to be undertaken and accelerates tree breeding targeted towards a specific gene [319]. The technique of the suppression of subtractive hybridization has been used to identify a transcription factor controlling fibre formation [320]. As they are controlling several genes related to one trait, transcription factors are ideal gene candidates for the improvement of fibre. Genetic engineering is another way to complement and improve conventional breeding [321]. To date, strategies for remodelling cell walls have focused on facilitating its digestibility in order to improve saccharification for biofuel production. They mainly target tightly-regulated endogenous factors, such as transcription factors, biosynthesis pathways, the nucleotide pool, polysaccharide synthases and surface mechano-sensors. [322,323,324,325]. Recently, for the same purpose, an elegant genetic engineering approach rewiring the secondary cell wall network of key genes and the creation of an artificial positive feedback loop [326] has been implemented.

### 8.3. Pulping Improvements

Fibre extraction has a strong influence on fibre quality [327]. Using biotechnology to facilitate extraction could reduce the cost of processing and improve the fibre quality by reducing fibre damage [239]. A target characteristic of the plant cell wall should be a high cellulose content and crystallinity, low level of lignification and cross linking [328]. Limiting lignin-hemicellulose-cellulose network linkages by modifying lignin content [329,330], pectin methylation [331], fucosyl substitution of xyloglucan [332,333], [Me]GlcA and acetylation substitution of xylan [334] or ferulic acid substitution of xylan [133] are other potential molecular targets for fibre modification. On the other hand, a high hemicellulose content is correlated with higher nanofibrillation of fibres and individualization of the microfibrils [335]. Thinner cell walls are easier to fibrillate and give higher yields of the nanofibrillated fraction [335]. Lastly, parameters, such as growing conditions [336] and the method of decortication [37], are known to have an impact on mechanical properties.

## 9. Conclusions

Plant fibres have been used as materials by humans for thousands of years because they had the mechanical properties and durability to fulfil our needs (ropes, cloth, etc.). However, their primary role, arising via evolution, is as a structural support and sap conduction element within a living plant. Therefore, plant fibres reach their full mechanical potential associated with each other in a hydrated state. When we extract them from a plant, we are fundamentally altering their properties by individualising, physically damaging, chemically altering and drying them. Even if they have good mechanical properties compared to synthetic fibres, their inherent hydrophilicity impairs them from achieving high performance in WPC. Nevertheless, there is an upsurge of interest for short natural fibre-reinforced polymer composites for applications, such as construction [337], the automotive industry [338] and aerospace [339]. This has mainly arisen after parameters, such as void content, the interface and interphase, have been improved. The future of natural fibre is bright [340], as there is a research emphasis on the cell wall molecular structure and genetic control, plant fibre chemical modification and green nanotechnology.
